# Sexual selection maintains whole-body chiral dimorphism in snails

**DOI:** 10.1111/j.1420-9101.2007.01370.x

**Published:** 2007-09

**Authors:** M. SCHILTHUIZEN, P. G. CRAZE, A. S. CABANBAN, A. DAVISON, J. STONE, E. GITTENBERGER, B. J. SCOTT

**Affiliations:** * Institute for Tropical Biology and Conservation, Universiti Malaysia Sabah Kota Kinabalu, Malaysia; † National Museum of Natural History ‘Naturalis’ Leiden, The Netherlands; ‡ School of Biological Sciences, University of Bristol Bristol, UK; § WWF-Malaysia Kota Kinabalu, Malaysia; ¶ Institute of Genetics, School of Biology, University of Nottingham Nottingham, UK; ** Department of Biology, McMaster University Hamilton, ON, Canada; †† Institute of Biology, Leiden University Leiden, The Netherlands; ‡‡ Sustainability Group, Victoria University Melbourne City, Vic., Australia

**Keywords:** *Amphidromus*, balanced polymorphism, chirality, Gastropoda, Malaysia

## Abstract

Although the vast majority of higher animals are fixed for one chiral morph or another, the cause for this directionality is known in only a few cases. In snails, for example, rare individuals of the opposite coil are unable to mate with individuals of normal coil, so directionality is maintained by frequency-dependent selection. The snail subgenus *Amphidromus* presents an unexplained exception, because dextral (D) and sinistral (S) individuals occur sympatrically in roughly equal proportions (so-called ‘antisymmetry’) in most species. Here we show that in *Amphidromus* there is sexual selection for dimorphism, rather than selection for monomorphism. We found that matings between D and S individuals occur more frequently than expected by chance. Anatomical investigations showed that the chirality of the spermatophore and the female reproductive tract probably allow a greater fecundity in such inter-chiral matings. Computer simulation confirms that under these circumstances, sustained dimorphism is the expected outcome.

## Introduction

Because of its ubiquity and binary nature, bilateral asymmetry is one of the few developmental characters that are suitable for comparative evolutionary studies in animals (Palmer, 2004). Although some species show antisymmetry, with dextral (D) and sinistral (S) morphs equally common, most species are directionally asymmetric, fixed for one morph or another (Palmer, 2005). The cause of directional asymmetry is clear in only a few groups (Vermeij, 1975), such as in gastropods (snails). Their coiling direction (not only of the shell but also of the entire body organization), determined by a single locus of maternal effect (Sturtevant, 1923; Diver *et al.*, 1925; Degner, 1952; Murray & Clarke, 1976), is usually fixed within a species because mating among D and S individuals (so-called ‘inter-chiral mating’) is either impossible or very difficult (Johnson, 1982; Asami *et al.*, 1998; Ueshima & Asami, 2003). Consequently, a rare reverse-coiled morph will normally not persist because of frequency-dependent selection (Gittenberger, 1988; Van Batenburg & Gittenberger, 1996; Schilthuizen & Davison, 2005).

The South-East Asian camaenid tree snail subgenus *Amphidromussensu stricto* is an intriguing and unexplained exception (Palmer, 1996; Schilthuizen & Davison, 2005; Schilthuizen *et al.*, 2005; Sutcharit *et al.*, 2006). It contains 35 species throughout South-East Asia, the majority of which (∼30 species) are always or almost always dimorphic, with D and S individuals occurring in the same population in roughly equal frequencies (Pilsbry, 1900; Laidlaw & Solem, 1961; Solem, 1983; Lehmann & Maassen, 2004; Craze *et al.*, 2006; Sutcharit *et al.*, 2006; Sutcharit & Panha, 2006a), although proportions are expected to deviate substantially from 1 : 1 under certain circumstances (P.G. Craze, unpublished data).

Several hypotheses have been proposed to explain this maintenance of dimorphism in *Amphidromus* (Laidlaw & Solem, 1961; Schilthuizen & Davison, 2005; Schilthuizen *et al.*, 2005; Craze *et al.*, 2006; Sutcharit *et al.*, 2006). First, metapopulation structure could lead to microspatial mosaics of monomorphism, producing dimorphism on a larger spatial scale. However, this has already been discounted as morphs have been found to coexist even at the smallest spatial scales (Schilthuizen *et al.*, 2005; Craze *et al.*, 2006; Sutcharit *et al.*, 2006). A second hypothesis is that frequency-dependent selection is counteracted by extrinsic balancing selection. For example, predators might specialize on the commoner morph (Dietl & Hendricks, 2006) until it becomes rare, at which point they would switch to the alternative morph. A third possible explanation is that the two morphs differ in traits that might allow them to occupy different niches. And a final explanation is that dimorphism is maintained by intrinsic idiosyncrasies of *Amphidromus* coiling genetics (e.g. offspring morph ratio is 1 : 1 irrespective of genotype) or reproduction.

In this article, we study a dimorphic population of one *Amphidromus* species, and evaluate support (or lack thereof) for each of the above-mentioned hypotheses. Eventually, using a combination of field observation, anatomy and computer simulation, we obtain strong indication that dimorphism is maintained by sexual selection.

## Material and methods

### Field procedures

*Amphidromus inversus* is common throughout the wholly-forested Kapas island (1.7 × 0.7 km). This population was, based on its pale shell colour, recently described as *A. inversus albulus* by Sutcharit & Panha (2006b); however, given the known variability in shell coloration, we here refrain from applying subspecific status. Living snails and empty shells were collected by hand at site 1 (5°12′55′′N, 103°15′49′′E; 160 × 300 m) and site 2 (5°13′22′′N, 103°15′45′′E; 240 × 70 m). The predation assessment was based on adult and juvenile shells; on the basis of the damage, rats were identified by experienced local mammalogists as the most likely predators (H. Bernard, G. Davison, J. Payne, & K. Wells, personal communication). We have no indication that other agents of predation are important in this population. Chi-squared tests for homogeneity and goodness-of-fit for morph frequencies were carried out manually (Zar, 1999).

### Husbandry

Egg clutches collected in the field were kept in bags made of nylon stocking and left to hatch in separate containers in dark, ambient conditions. Although we have been able to maintain adults in captivity and let them produce offspring, we have not yet been successful in rearing them from egg to adulthood. Consequently, we could not determine which chirality allele is dominant.

### Anatomy

Fifty-eight adult snails were killed in deoxygenated water and fixed in 70% ethanol. Ten mating pairs were snap-frozen with electrical component freezing spray (Craze & Barr, 2002) and immediately fixed in 70% ethanol. The entire reproductive system of all 78 snails was dissected, and in some cases cleared with clove oil. The bursa copulatrix was opened and the coiling direction of any spermatophore fragments was noted. A conservative approach was adopted to use these data for the inference of copulations, e.g., if a sinistral spermatophore was found in a dextral individual, and a dextral one in a sinistral individual in the same sample, they were assumed to have resulted from a single copulation. To ascertain that the opening of the spermatophore is situated at the terminal end of the ‘coiled expanded section’ (CES; see also Sutcharit & Panha, 2006a), dye was pushed through the lumen using a syringe.

### Morphometrics

Ten shell traits were measured in 100 randomly selected adult shells (50 D and 50 S, each with equal representation of sites 1 and 2) with a pair of Vernier callipers to the nearest 0.05 mm: shell height; shell width; body whorl width; aperture height; aperture width; lip width; apertural height of penultimate whorl; apertural height of second-last whorl; apertural height of third-last whorl; angle between apertural surface and columella. The untransformed measurements were subjected to a principal component analysis (PCA) in SPSS using varimax rotation. We chose PCA rather than manova, because PCA allowed us to separate size (in component 1) from shape (in the remaining components). The first two components extracted 81% of variance. Seventy-one per cent of correlations lay between 0.30 and 0.75. Bartlett's test of sphericity was significant (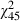 = 1154, *P* < 0.001) and the Kaiser-Meyer-Olkin measure of sampling adequacy was 0.904. Thus, the conditions for carrying out a PCA were met.

### Simulated random mating

The expected frequencies of pairs under random mating could not be calculated in a simple fashion due to the tree-based population structure. For this reason, they were simulated using random pairing. The numbers and morph ratios for 92 trees were recorded in 2003 (Schilthuizen *et al.*, 2005). We used these as a basis to simulate the expected morph composition of a group of 80 each year for 3 years; that is, removing trees with only one snail, as in the absence of selfing such trees would not contribute to the number of pairs. Numbers on the trees were kept constant but the proportion of dextrals on each tree was modelled as a random, normally distributed variable with mean equal to the observed proportion of dextrals on that tree and variance calculated assuming a normal approximation to a binomial. For each of the 3 years we then estimated the proportion of DD, DS and SS pairs as follows. To form the pairs we assumed a snail was drawn at random from those available. Its partner was then drawn at random from those remaining on the tree. Provided the number of pairs in each year was not too large we then estimated the expected proportions of pairs of the three kinds as follows: 
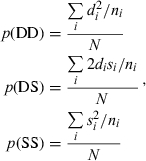
 where *p*(XY) is the expected proportion of XY pairs, *d*_*i*_ is the number of dextral snails on tree *i*, *s*_*i*_ is the number of sinistral snails on tree *i*, *n*_*i*_ is the total number of snails on tree *i* and *N* is the total number of snails on all 80 trees. After the 3 years we compared the expected numbers of pairs with those observed and calculated a chi-squared statistic. The whole procedure was repeated 10 000 times and the figures reported below are the mean and 95% confidence limits derived from the 10 000 repeats.

### Model

A simulation model was necessary because the delayed inheritance of the coiling gene results in non-obvious population genetic behaviour (Kirkpatrick & Lande, 1989; Lande & Kirkpatrick, 1990). The basic structure of the model was one of discrete, non-overlapping generations with the population maintained at the calculated neighbourhood size of 208 [determined following Wright (1969) and Cowie (1984) using data in Schilthuizen *et al.* (2005)]. These 208 individuals were divided between 52 trees, with an average of four snails per tree, the closest integer to the mean number observed in *A. inversus* (Schilthuizen *et al.*, 2005). Each generation experienced a mating season of 120 days, set at this length after considering the timing of suitable weather conditions (Malaysian Meteorological Service, 2005).

The sequence of events occurring on each day of the mating season was as follows. Snails first had the opportunity to move between trees. The probability of an individual moving to any of the other 51 trees was set at 0.03, the observed daily probability of a marked *A. inversus* being found on a tree other than the one on which it was marked (Schilthuizen *et al.*, 2005). Then, for each tree independently, pairs of snails were randomly selected. A pair mated with a probability that varied with the day of the mating season (*day*) and the phenotypes of the two partners. The overall probability of mating was a logistic function of the mating season day: 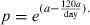
(1)

**Fig. 1:**
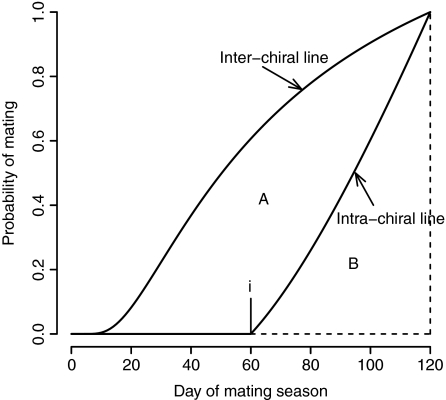
Probability curves for inter- and intra-chiral encounters proceeding to mating and the areas involved in the calculation of the disassortative mating index. Area *A* is that between the two curves whereas *B* is the area under the intra-chiral curve. The probability of an intra-chiral mating occurring is set at zero if *day* is less than *i*.

For reasons given below, *a* was set to 0.5. Where disassortative mating was investigated, the probability of an intra-chiral mating was reduced relative to that for an inter-chiral mating by multiplying *p* by a factor (*q*) that was a simple linear function of the mating season day: 
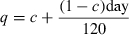
(2) where *c* is a constant. Eqn 2 was constrained to equal zero where a negative probability would result, and to equal 1 on the last day of the mating season. A pair was therefore certain to mate if they survived to the end of the mating season (see below) and were on the same tree. To determine the value of *c* we first defined a disassortative mating index with reference to Fig. 1. This was the ratio of the area between the inter- and intra-chiral mating probability curves (*A*) to the total area under the inter-chiral curve (*A* + *B*). So, when the two curves are equal, the area between them is zero and the disassortative mating index is zero. When there is complete disassortative mating, the intra-chiral mating curve follows the *x*-axis and so the area between the curves is equal to the total area under the inter-chiral curve, giving a disassortative mating index of 1.00. We then specified the parameters of Eqn 2 by setting values of the disassortative mating index to generate the area *B* and then iteratively solving the following integral for *c* and *i*: 
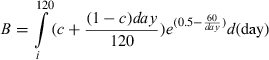
(3)

**Fig. 2:**
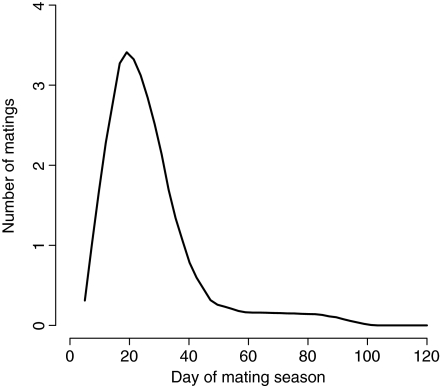
Smoothed total number of matings observed against *day* for a typical day of the mating season. After the burn-in period, the appearance of the curve was similar for different values of the model parameters and varied little through the breeding season or from one generation to the other.

Neither the choice of 0.5 for the parameter *a* nor the use of a linear function for the intra-chiral adjustment (*q*) was arbitrary. We were guided by expectations based on our own field experience and the small amount of literature on the subject (Emberton, 1985; Madec & Daguzan, 1993; Giokas & Mylonas, 2002; Parmakelis & Mylonas, 2002). The functions and parameters we chose gave the expected behaviour (Fig. 2).

If an encounter did not proceed to mating, the program continued to search through all other unique pairwise combinations of snails on the focal tree. Otherwise, offspring were generated. We assumed the genetic basis of chirality in *Amphidromus* to be the same as in all other chiral species so far studied; i.e. a single locus with two alleles and maternal inheritance. It is not known whether sinistrality or dextrality is dominant in this genus and, as the results do not depend on this, we arbitrarily made the sinistral allele dominant. We produced offspring based on the usual combinations from a diploid locus but with half expressing the phenotype appropriate to the first maternal genotype and the other half expressing the second. We set the baseline number of offspring per individual to 20 (the average number among 36 egg batches observed in the field), which means each pair produced 40 offspring. To allow for fecundity differences between inter- and intra-chiral pairs, we increased the number of offspring from inter-chiral matings by randomly sampling from the 40 offspring produced. So, if, for example, the relative fecundity was 1.05, we sampled two offspring from the 40 to give a total of 42 (or 1.05 × 40).

When two snails mated they were removed from the population. The program worked through all 52 trees in turn with only those snails that had not yet mated moving on to the next day of the breeding season. At this stage the surviving snails suffered mortality at a constant daily rate of 1.897 × 10^−4^. This value was calculated using the field observation that roughly half of the population of *A. inversus* survives from one year to the next (Schilthuizen *et al.*, 2005). We then estimated the daily rate of mortality (*m*) assuming this to be constant (in the absence of additional prior information) and using the re-arranged binomial expression: 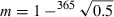
(4)

After the application of mortality, the surviving snails repeated the daily cycle of movement between trees, encounter, mating and mortality until the end of the breeding season when any that had not mated were removed. The large number of offspring was reduced to the neighbourhood size of 208 by random sampling and the number of snails per tree adjusted by randomly assigning individuals from trees with more than four snails to trees with fewer than four. The population had then passed through a generation and all breeding season processes were repeated. The population passed through 2510 generations, which included a 10-generation burn-in period to remove effects of the starting population (a heterozygous population with equal numbers of phenotypically sinistral and dextral snails on each tree).

## Results and discussion

**Fig. 3:**
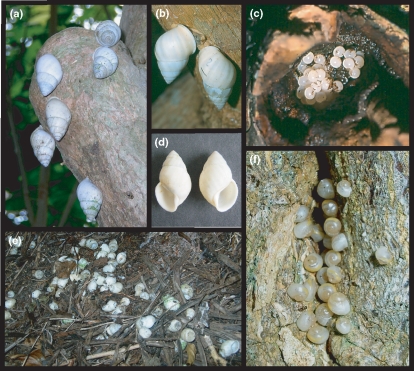
*Amphidromus inversus* on Kapas island, Malaysia. (a) One dextral (top) and five sinistral adults (approximate shell height: 40 mm); (b) inter-chiral copulation between a dextral (left) and a sinistral (right) individual; (c) a freshly laid clutch of eggs; (d) a sinistral (left) and a dextral (right) adult shell; (e) a large number of apically crushed shells in a rat midden; (f) a recently hatched egg clutch, all sinistral (mean shell diameter: 3 mm).

The population of *A. inversus* (Fig. 3d) on the Malaysian island of Kapas (1.25 km^2^) that we studied occurs in trees in primary and coastal forest at densities of about 1000 adults per hectare (Fig. 3a); the numbers of adults per tree range from 0 to 60; no morph clustering can be detected at any spatial scale; and dispersal is equal in both morphs (Schilthuizen *et al.*, 2005). We chose two localities (sites 1 and 2), separated by 640 m. In both places, we found that D : S proportions were identical (frequency of D [*d*] ∼0.35) in old, eroded shells from the forest floor, and in the 2003 and 2005 live adult populations (Table 1a). This suggests that dimorphism is stably maintained both spatially and temporally.

**Table 1:** Dextral (D) and sinistral (S) morph frequencies in (A) adults and (B) shells with predation marks.

	Site 1			Site 2		
		
	D	S	d*	D	S	d*
(a) Morph frequency						
Old eroded shells	488	798	0.379†	308	602	0.338†
2003 live adults	232	417	0.357†	76	144	0.345†
2005 live adults	86	155	0.356†	92	176	0.343†
(b) Predation frequency						
Total empty shells	415	674		212	440	
Traces of predation	111	197		56	117	
Predation frequency	0.267‡	0.292‡		0.264‡	0.266‡	

* Frequency of dextrals.

† All frequencies are homogeneous (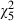 = 4.535, *P* = 0.475) and not significantly different from *d* = 0.35 (pooled samples: *χ*^2^_1_ = 1.190, *P* = 0.275).

‡ All frequencies are homogeneous (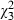 = 1.421, *P* = 0.701) and not significantly different from 0.275 (pooled samples: *χ*^2^_1_ = 0.014, *P* = 0.905).

We then looked for traces of predation by rats (the snails’ chief predators) in empty, fresh shells at both localities (Fig. 3e). Total predation frequencies were identical (∼0.275) for both morphs in both localities (Table 1b), whereas a higher predation frequency would be expected in the commoner (sinistral) morph in the case of a predator bias. We therefore conclude that as in other *Amphidromus* species (Craze *et al.*, 2006) predation is not frequency-dependent. Moreover, dimorphic *Amphidromus* species occur in many different habitats from India to the Moluccas, and it is likely that their range of natural enemies will be too variable for a predation-related explanation to be generally applicable [see also Sutcharit *et al.* (2006) for further criticism of the predation hypothesis].

Subsequently, we examined shell shape by measuring 10 biometric variables and extracting two principal components. There was no significant clustering of D and S shells along either of the axes (*t*_98_ = −0.051, *P* = 0.96 for PC1; *t*_98_ = 1.28, *P* = 0.20 for PC2). This confirms that both morphs are mirror images of each other, which may be considered as an indication that the forms are ecologically equivalent. Combined with our earlier evidence (Schilthuizen *et al.*, 2005) that they are identical in terms of dispersion, dispersal and habitat preference, and molecular data showing that dimorphic *Amphidromus* populations are fully panmictic (Sutcharit *et al.*, 2006), we do not have any indication that niche differences exist between the morphs.

Finally, to investigate the inheritance of coiling, we collected eight egg batches (4–70 eggs each; Fig. 3c) laid by single snails and let these hatch. We also found 28 clutches of 3–57 freshly-hatched juveniles in the field (Fig. 3f). In all cases except one, the entire clutch was invariant for coiling direction. This is consistent with chirality being caused by a single locus of maternal effect, as in other snails: hatchlings coil in the direction dictated by their mother's genotype (Sturtevant, 1923; Diver *et al.*, 1925; Degner, 1952; Murray & Clarke, 1976; Schilthuizen & Davison, 2005; Sutcharit *et al.*, 2006). The one exception was an egg batch that yielded 23D and 2S. Similar rare mixed broods have been found in other species as well (Schilthuizen & Davison, 2005), although in this case the eggs were collected from the wild, and the result may be due to oviposition by two mothers in the same place.

The above shows that dimorphism is stable, but cannot be explained by population structure, extrinsic balancing selection, niche differentiation or unusual coiling genetics. However, we did find evidence for an unexpected mating behaviour.

Following Davison *et al.* (2005), we use *α* to denote the degree to which inter-chiral copulation is inhibited. In hermaphroditic snails with globular shells and face-to-face mating, such as *Helix*, *Bradybaena* and *Euhadra*, inter-chiral copulation is impossible due to incompatible positions of the genital openings, and *α* = 1.00 (Meisenheimer, 1912; Gittenberger, 1988; Asami *et al.*, 1998; Ueshima & Asami, 2003; Schilthuizen & Davison, 2005). In high-spired snails, which mate by shell-mounting, however, inter-chiral copulation is usually possible, but still significantly impeded. In *Partula suturalis*, for example, *α* = 0.74 (Johnson, 1982; Schilthuizen & Davison, 2005).

In field observations of *A. inversus* mating, we found that they are unusual in that they have a high-spired shell, but mate face-to-face (Fig. 3b). Mating was concentrated at the onset of the monsoon season (August–December). Copulation, preceded by up to 30 min of courtship during which the animals circle (and sometimes reject) each other, took place during daytime and normally lasted for 6–9 h, irrespective of their coiling directions. It was always simultaneous and reciprocal, with both hermaphroditic partners donating and receiving a spermatophore at the same time (see Material and methods).

Throughout three mating seasons (2003–2005), we recorded the chirality of 85 copulating pairs. We also dissected the ‘spermatophore receiving organ’ (SRO; Koene & Schulenburg, 2005) of 78 ethanol-preserved adults that were collected in the 2003 and 2005 mating seasons. As the coiling direction of the donor can be determined from the shape of the spermatophore (see below), this allowed for the inference of chirality of a further 21 pairs (the SRO in many individuals was empty). Overall, at site 1, we found 13 D × D, 42 D × S and 25 S × S pairs, and at site 2, five D × D, 14 D × S and seven S × S pairs. As morph frequencies were constant across years and localities (see above), we pooled these data to give 18 D × D, 56 D × S and 32 S × S pairs.

Surprisingly, there was a highly significant *excess* of inter-chiral mating, both at each site separately, and using the pooled data (*χ*^2^_2_ = 17.7; *P* = 0.0002; based on simulated random mating). Using the morph frequencies among the 106 pairs, random mating would give lower and upper 95% confidence limits of 34.6 and 37.9 D × S pairs respectively. Consequently, *α* must be considered to range between −0.38 and −0.32 in this species, although it is as yet unclear exactly how *A. inversus* overcomes the mechanical obstacles to inter-chiral mating. As all computer simulations of the population genetics of snail coiling so far have been carried out with *α* ≥ 0 (Johnson *et al.*, 1990; Orr, 1991; Van Batenburg & Gittenberger, 1996; Stone & Björklund, 2002; Schilthuizen & Davison, 2005), we created a computer model that simulates the *A. inversus* population under a range of negative values for *α*. Due to the delayed inheritance, the effects of negative *α* on the stability of dimorphism are not trivial (Kirkpatrick & Lande, 1989; Lande & Kirkpatrick, 1990), and cannot be predicted without the model.

**Fig. 4:**
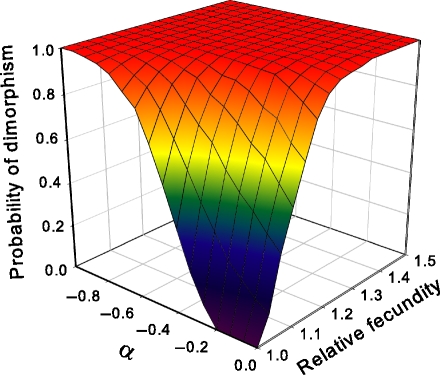
Results from the computer simulation of the *Amphidromus inversus* population, showing the probability of dimorphism using a range of values for inter-chiral mating failure (*α*) and inter-chiral relative fecundity. Values of *α* < 0 indicate an excess of inter-chiral mating. Values of the fecundity ratio > 1.00 indicate the proportionate increase in the number of offspring produced after inter-chiral mating. Probability of dimorphism is defined as the proportion of runs with a given set of parameter values that remained dimorphic after 2510 generations.

Our simulations show that S snails are approximately three times more likely to carry the allele for sinistrality than are D snails. This means that there is a considerable correlation between genotype and phenotype, in spite of the delayed inheritance. In accordance with this genotype–phenotype correlation, the simulations show that negative values for *α* are sufficient to stabilize the dimorphism in 10–40% of the cases within the probable range for *α* of −0.30 to −0.40 (Fig. 4, data points for relative fecundity = 1.0). A study of the reproductive anatomy (below) explains why the snails may prefer the normally disadvantageous inter-chiral mating, and how this may further stabilize the dimorphism.

**Fig. 5:**
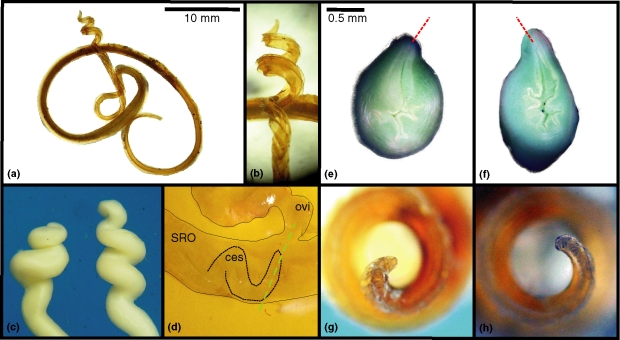
*Amphidromus inversus* reproductive anatomy. (a) An entire sinistral spermatophore; (b) the coiled expanded section (CES) of a sinistral spermatophore; (c) the epiphallic caeca (which produce the CES) of a sinistral (left) and a dextral (right) individual; (d) the CES of a recently deposited sinistral spermatophore at the junction of the spermatophore receiving organ (SRO) and the free oviduct (ovi) of a dextral individual (outlines added for clarity; the green line indicates the plane of the sections in e and f); (e, f) vaginal view of the entrance of the free oviduct in a dextral (e) and a sinistral (f) individual; the red arrows indicate the direction of attachment of the oviduct, which corresponds with the coil of the spermatophore tips (g, h) in an inter-chiral mating; (g, h) apical view of the CES in a sinistral (g) and a dextral (h) spermatophore.

We found that the CES of the 60-mm-long spermatophore carries three coils (Fig. 5a,b); these coils (and those in the epiphallic caecum that produces the spermatophore tail; Fig. 5c) follow the coiling direction of the shell: they are dextral in dextral snails and sinistral in sinistral snails. Dissection of snap-frozen copulas showed that when the spermatophore is deposited in the SRO, the tip of the CES, which releases spermatozoa, is situated at the entrance to the free oviduct (Fig. 5d). The oviduct is connected with the SRO at an angle to the left or to the right in a sinistral or a dextral recipient, respectively, whereas the spermatophore tail tip points to the left or right in a dextral or a sinistral donor respectively (viewed from the front of the recipient animal). Consequently, it can enter the oviduct more easily in inter- than in intra-chiral copulation (Fig. 5e–h).

Because in snails the SRO has evolved to digest the vast majority of sperm (Koene & Schulenburg, 2005), it is likely that if proportionately more sperm escape into the oviduct then proportionately more of them will fertilize the eggs. A snail's tendency to mate with the opposite coiling morph is therefore likely to have arisen as a result of the consequent increase in fecundity. Although increased inter-chiral fecundity is deduced on the basis of anatomy, rather than proven experimentally, our further simulations show that it can contribute to stable dimorphism. Simulation with negative values for *α* and increased inter-chiral fecundity shows that a large parameter space exists where dimorphism is stably maintained (Fig. 4), and that, with the observed *α*, stability is always expected if inter-chiral fecundity rises by more than 10%.

Hence, our results show that sexual selection for inter-chiral mating, combined with an increase in inter-chiral fecundity, is the most likely explanation for the maintenance of dimorphism in *A. inversus*. As the literature shows that all other anatomically studied species of *Amphidromus* s. str. also have a coiled epiphallic caecum, the explanation is likely to be a general one. In support of our conclusion is the fact that the > 50 species in the sister subgenus, *Syndromus*, which has a very short, non-coiled epiphallic caecum (Pilsbry, 1900; Sutcharit & Panha, 2006a), are never dimorphic.

In conclusion, we have shown that mutual mate choice for a partner with mirror-image asymmetry stabilizes antisymmetry in these snails. As far as we are aware, this is the first confirmed case of heritable antisymmetry in the Metazoa (Palmer, 2005). It is interesting that the best characterized case of plant antisymmetry has also arisen out of sexual selection (Jesson & Barrett, 2002). Chirality occurs in primary sexual traits in many other animal groups, e.g. cephalopods, insects and fish (Balderson, 1978; Palmer, 1996, 2004, 2005; Spéder *et al.*, 2006). Our study shows that during phylogenesis, directional asymmetry may become unstable and pass through a stage of stable dimorphism because of sexual selection. This may help explain the phylogenetic transitions in chirality throughout the Metazoa.
